# Analysis of causes and outcomes of principal secondary thrombotic microangiopathy: a 9-year cohort study from a tertiary pediatric center in China

**DOI:** 10.1016/j.rpth.2026.106850

**Published:** 2026-07-14

**Authors:** Dan Wu, Jie Zheng, Jun Yang, Bin Wang, Guanghua Zhu, Zhi Chen, Qiang Sun, Nan Zhou, Zhenping Chen, Yanhui Luo, Wei Yang, Xiaoyu Tian, Hejia Zhang, Xue Liu, Lei Lei, Na Tian, Kaige Zhang, Li Fang, Lan Mi, Ang Wei, Ying Liang, Maoquan Qin, Xiaorong Liu, Chenguang Jia

**Affiliations:** 1 Department of Nephrology, National Center for Children's Health, Beijing Children's Hospital, Capital Medical University, Beijing, China; 2 Department of Stem Cell Transplantation, Hematology Center, National Center for Children's Health, Beijing Key Laboratory of Pediatric Hematology Oncology, National Key Discipline of Pediatrics (Capital Medical University), Key Laboratory of Major Diseases in Children, Ministry of Education, Beijing Children's Hospital, Capital Medical University, Beijing, China; 3 Clinical Laboratory, Beijing Children’s Hospital, Capital Medical University, Beijing, China

**Keywords:** hemolytic-uremic syndrome, child, cohort study, thrombotic microangiopathy, thrombotic thrombocytopenic purpura

## Abstract

**Background:**

Few large-scale cohort studies have reviewed consecutive cases of thrombotic microangiopathy (TMA), especially in children.

**Objectives:**

The aim of our study was to evaluate causes and outcomes of TMA at a tertiary pediatric center in China over a 9-year period.

**Methods:**

We retrospectively analyzed consecutive pediatric TMA patients admitted to Beijing Children's Hospital from December 2015 to January 2025. The primary endpoint was death or progression to kidney failure. Multivariable Cox regression identified independent predictors of this composite endpoint.

**Results:**

We retrospectively analyzed 303 consecutive pediatric patients with a first episode of TMA. Secondary TMA was the most common type (*n* = 172; 57%), followed by atypical hemolytic uremic syndrome (*n* = 93; 31%), thrombotic thrombocytopenic purpura (*n* = 24; 8%), and Shiga toxin-producing *Escherichia coli*-associated hemolytic uremic syndrome (*n* = 14; 5%). Among the 172 cases of secondary TMA, the most common causes were hematopoietic stem cell transplantation (*n* = 95; 55%) and systemic diseases (*n* = 40; 23%). After a median follow-up of 35 months, death or kidney failure occurred in 53 (31%), 7 (8%), 1 (4%), and 0 patients with secondary TMA, atypical hemolytic uremic syndrome, thrombotic thrombocytopenic purpura, and Shiga toxin-producing *Escherichia coli*-associated hemolytic uremic syndrome, respectively. Multivariate Cox regression showed that secondary TMA (hazard ratio [HR], 3.57; 95% CI, 1.53-8.30; *P* < .01), the presence of heart failure and/or shock at onset (HR, 2.21; 95% CI, 1.24-3.93; *P* = .01), and the need for mechanical ventilation support due to respiratory failure (HR, 3.88; 95% CI, 2.19-6.88; *P* < .001) were independent risk factors for death or kidney failure.

**Conclusion:**

This study shows that secondary TMA is the predominant form of pediatric TMA. Secondary TMA, heart failure/shock, and the requirement for mechanical ventilation were identified as independent risk factors for death or kidney failure in children.

## Essentials

• Large-scale studies evaluating the overall disease spectrum of consecutive pediatric TMA patients are lacking.
• This study reports on a long-term cohort study evaluating the causes and outcomes of pediatric TMA in China.
• Secondary TMA was the predominant cause of pediatric TMA and a key independent risk factor for death or kidney failure.
• Heart failure/shock and the requirement for mechanical ventilation at onset were independent risk factors for death/kidney failure.

## Graphical abstract

**Figure:**
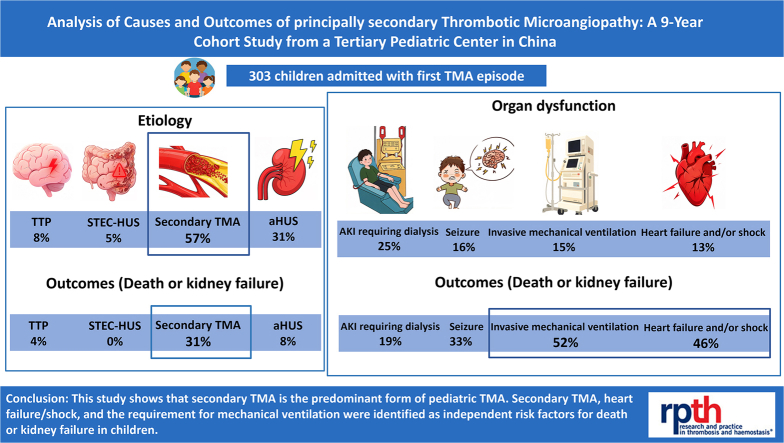

## 1 Introduction

Thrombotic microangiopathy (TMA) is an extremely rare and life-threatening group of disorders caused by endothelial injury from various etiologies. It is characterized by a triad of microangiopathic hemolytic anemia, thrombocytopenia, and potential end-organ damage or pathologic evidence of microvascular injury and thrombosis [[1], [2], [3]]. Based on underlying mechanisms, TMA is classified into thrombotic thrombocytopenic purpura (TTP), atypical hemolytic uremic syndrome (aHUS; also known as complement-mediated TMA), Shiga toxin-producing *Escherichia coli*-associated hemolytic uremic syndrome (STEC-HUS), and TMA associated with specific diseases (also known as secondary TMA) [2,4].

Advances in our understanding of the potential mechanisms of TMA have spurred the development of targeted therapies, leading to better outcomes for patients with TTP and aHUS. TTP is caused by a deficiency in the activity of the vascular von Willebrand factor-cleaving protease (a disintegrin-like and metalloprotease with thrombospondin type 1 motif 13 [ADAMTS-13]), leading to persistent ultralarge von Willebrand factor multimers, platelet activation, and microvascular thrombosis [5]. Two main forms of TTP are immune TTP (iTTP) and congenital TTP (cTTP). At present, effective treatment for iTTP relies on a combination of plasma exchange, immunosuppression, and caplacizumab [6,7]. In addition, recombinant ADAMTS-13 is used to treat cTTP and severe iTTP [8]. STEC-HUS is caused by STEC infection and is diagnosed by detecting Shiga toxin in STEC cultures from patient stool samples and a polymerase chain reaction assay [9]. At present, management of STEC-HUS is supportive care; in most cases, plasma exchange and eculizumab are not required, and the effectiveness of antibiotics remains controversial [[10], [11], [12]]. aHUS is a complement-mediated disease characterized by hereditary or acquired defects in the alternative pathway of the complement system [1,13]. In patients with aHUS, inhibiting complement activation by using C5 monoclonal antibodies can greatly reduce the progression to kidney failure or death and prevent recurrence after kidney transplantation [9,14,15]. Secondary TMA includes many conditions or diseases associated with its development, such as solid organ or hematopoietic stem cell transplantation (HSCT), autoimmune disease, infections, drugs, malignant hypertension, and malignant tumors [[16], [17], [18], [19], [20]]. The mechanisms of secondary TMA are largely unknown and usually multifactorial, and the management of these patients is not well standardized.

STEC-HUS has long been considered the most common type of TMA in children [21,22]. In adult patients, however, secondary TMA induced by malignancies, transplantation, pregnancy, systemic diseases, and other causes consistently accounts for a significant proportion [23,24]. With improvements in public health and food safety, as well as increased awareness and diagnostic vigilance for TMA, the spectrum of pediatric TMA appears to be changing, particularly in tertiary medical centers. Pediatric HSCT and systemic diseases, as important categories of secondary TMA, have seen an increasing number of reports and studies on their underlying mechanisms in recent years [19,[25], [26], [27]]. Nevertheless, knowledge of their underlying mechanisms remains limited. Large-scale studies of the overall disease spectrum in consecutive pediatric TMA patients are still lacking (Supplementary Table S1). Existing pediatric studies have several limitations: some focus exclusively on a single subtype, such as aHUS cohorts; some lack long-term follow-up data; and some do not adequately include high-risk populations related to HSCT [[28], [29], [30]]. Therefore, the relative proportions, clinical characteristics, and prognosis of different TMA subtypes in consecutive pediatric cohorts have not yet been fully clarified. This knowledge gap limits further optimization of risk stratification and clinical management strategies for pediatric TMA.

Here, we conducted a 9-year retrospective cohort study that consecutively included 303 children with a first episode of TMA at a national tertiary pediatric center in China. This center regularly manages a substantial number of pediatric patients at high risk for TMA, notably those who have undergone HSCT and those diagnosed with systemic diseases. The aims of this study were to: 1) clarify the true etiologic spectrum of pediatric TMA cases at this center; 2) evaluate and compare the long-term prognosis of children with TMA across different etiologies; and 3) analyze independent risk factors for the composite endpoint of death or progression to kidney failure.

## 2 Methods

### 2.1 Study design, participants, and adjudication

This descriptive retrospective study included pediatric patients aged <18 years with a first episode of TMA who were consecutively hospitalized at the National Children’s Medical Center, Beijing Children’s Hospital, from December 2015 to January 2025. We searched the electronic medical records of Beijing Children’s Hospital using the following 4 International Classification of Diseases, 10th Revision diagnostic codes: M31.100 (TMA), M31.101 (TTP), D59.300 (HUS), and D59.403 (microangiopathic hemolytic anemia). Medical records were analyzed by physicians trained in nephrology and hematology to adjudicate patients. First, TMA diagnosis was verified using the following diagnostic criteria: (1) hemoglobin < 100 g/L, peripheral blood smear showing schistocytes, and lactate dehydrogenase >295 U/L; platelet count < 150 × 10^9^/L; with or without organ damage of varying severity; (2) kidney biopsy confirming that the condition is consistent with TMA. The exclusion criteria were as follows: multiple hospitalizations for the same condition; not the first diagnosis or admission during the recovery period. A strict hierarchical process was used to identify the cause of TMA as follows: (1) TTP was identified based on ADAMTS-13 activity < 10% [3]; (2) STEC-HUS was identified by a positive stool culture for *E coli* or a positive stool polymerase chain reaction, supported by the presence of fecal leukocytes or red blood cells on microscopy. If the patient presented with clear prodromal bloody diarrhea followed by typical disease progression, notably acute kidney injury after diarrhea, along with rapid clinical improvement with supportive care, a clinical diagnosis remained reasonable even in the absence of positive pathogen testing. (3) Secondary TMA was identified based on the presence of any other disease or condition associated with TMA. In line with current guidelines [2,4], patients with evidence of TMA were considered to have aHUS if TTP, STEC-HUS, or secondary TMA were absent.

The study endpoint was death or progression to kidney failure during the follow-up period, which ended in June 2025. Kidney failure was defined as the initiation of kidney replacement therapy, including dialysis or kidney transplantation. This study was conducted ethically in accordance with the World Medical Association Declaration of Helsinki. The study was registered on the Chinese Clinical Trial Registry (ChiCTR2500114441; chictr.org.cn), and the study protocol was approved by the Ethics Committee of the Beijing Children’s Hospital (reference number [2025]-Y-091-D). Informed consent was waived due to the retrospective study design.

### 2.2 Data collection and definitions

Demographic, clinical, and biological characteristics were obtained from the patients' medical records. The worst biological values within the first week of TMA diagnosis were recorded. Organ involvement was defined as follows: kidney involvement, defined as acute kidney injury based on creatinine criteria in line with the Kidney Diseases International Guidelines Outcomes guidelines [31]; gastrointestinal involvement, defined as diarrhea, upper or lower gastrointestinal bleeding, or abdominal pain; central nervous system involvement, defined as seizures, altered consciousness, visual disturbances, ataxia, or unsteady gait; respiratory involvement, defined as shortness of breath or difficulty breathing, pleural effusion, pulmonary edema, or pulmonary hemorrhage; cardiovascular involvement, defined as heart failure and/or shock, pericardial effusion, or ventricular hypertrophy.

### 2.3 Statistical analysis

Data were analyzed using SPSS v.31 (IBM, SPSS Inc). Results were presented as mean ± SD for normally distributed continuous variables, median and IQR for nonnormally distributed continuous variables, and number and percentage for categorical variables. Data were analyzed using Student’s *t*-test; analysis of variance followed by the least significant difference multiple comparison test, Kruskal–Wallis tests followed by multiple comparisons with Bonferroni correction, chi-squared tests, or Fisher’s exact test for comparisons, as appropriate. Survival time was defined as the interval from TMA diagnosis to the occurrence of the study endpoint. We performed a time-to-event analysis using Kaplan–Meier estimates and the log-rank test. In the time-to-event analysis, participants lost to follow-up were right-censored on the date of their last known follow-up without the event of interest. To identify independent risk factors for poor prognosis, a multivariate Cox proportional hazards model was constructed. Potentially relevant clinicopathologic predictors identified in the univariate analysis (*P* < .1) were included in the multivariate model. The results were presented as hazard ratios (HRs) and 95% CIs. All *P* values were 2-sided, with *P* < .05 considered statistically significant.

## 3 Results

### 3.1 Study population and etiologies of TMA

**Figure 1:**
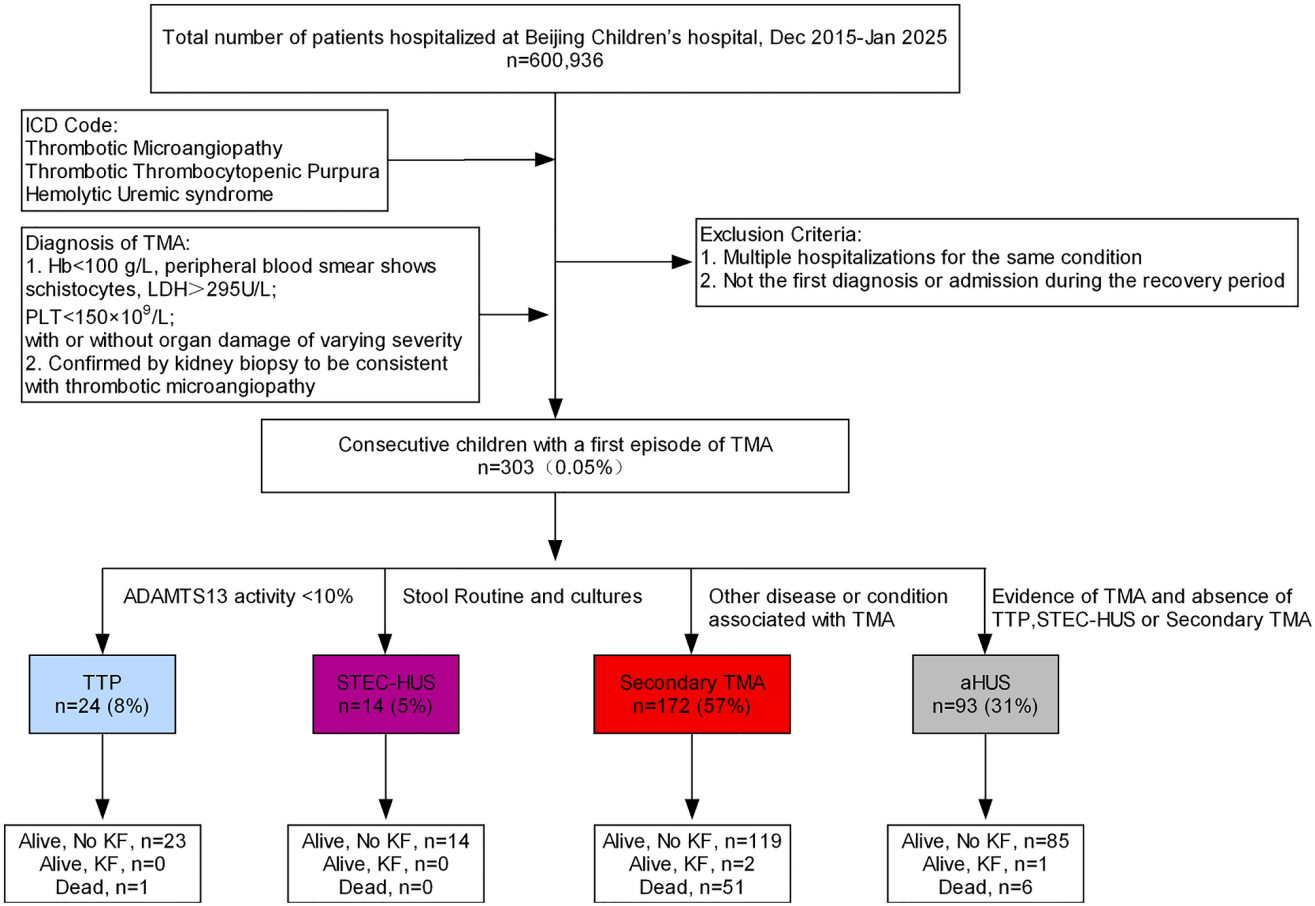
Flow chart of the study. ADAMTS-13, a disintegrin-like and metalloprotease with thrombospondin type 1 motif 13; aHUS, atypical hemolytic uremic syndrome; Hb, hemoglobin; ICD, International Classification of Diseases; KF, kidney failure; LDH, lactate dehydrogenase; PLT, platelet; STEC-HUS, Shiga toxin-producing *Escherichia coli*-associated hemolytic uremic syndrome; TMA, thrombotic microangiopathy; TTP, thrombotic thrombocytopenic purpura.

**Figure 2:**
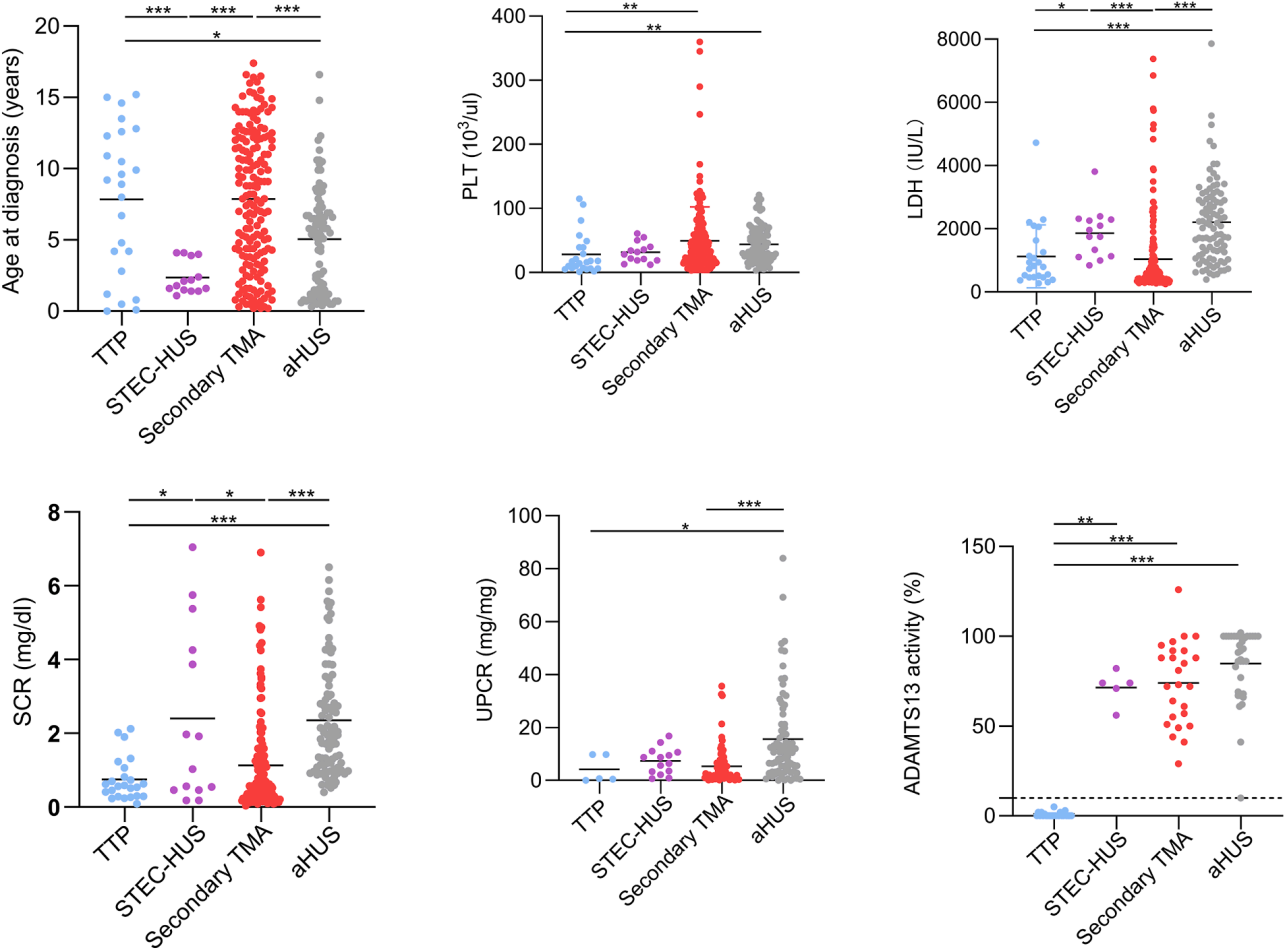
Baseline characteristics according to the etiology of thrombotic microangiopathy (TMA). ADAMTS-13, a disintegrin-like and metalloprotease with thrombospondin type 1 motif 13; aHUS, atypical hemolytic uremic syndrome; LDH, lactate dehydrogenase; PLT, platelet; SCR, serum creatinine; STEC-HUS, Shiga toxin-producing *Escherichia coli*-associated hemolytic uremic syndrome; TTP, thrombotic thrombocytopenic purpura; UPCR, urine protein-to-creatinine ratio. ∗*P* < .05; ∗∗*P* < .01; ∗∗∗*P* < .001.

We screened a total of 600,936 patients hospitalized at a single large pediatric reference center in China over 9 years and identified 303 consecutive children who experienced a first episode of TMA (0.05%; Figure 1). The distribution of TMAs by etiology was as follows: TTP in 24 (8%) cases, STEC-HUS in 14 (5%) cases, secondary TMA in 172 (57%) cases, and aHUS in 93 (31%) cases (Figure 1 and Table 1). The causes of secondary TMA were diverse, mainly including HSCT (95/172, 55%), systemic diseases (40/172, 23%), cobalamin C deficiency (16/172, 9%), infection (12/172, 7%), malignant hypertension (7/172, 4%), and neoplasia (2/172, 1%; Supplementary Table S2). Among the malignant hypertension cases, etiologies included renal artery stenosis (*n* = 2), renal dysplasia (*n* = 2), and idiopathic cases (*n* = 3), in which no specific cause was identified despite a comprehensive evaluation. The median age of children at admission was 6 years (IQR, 2-11), and 154 (51%) were male. STEC-HUS occurred only in children aged <5 years. aHUS showed a bimodal age distribution, peaking in children aged <2 years (29/93, 31%) and 5 to 8 years (52/93, 56%). TTP and secondary TMA occurred across all pediatric age groups, with median ages of 9 years (IQR, 3-13) and 8 years (IQR, 4-12), respectively (Figure 2 and Table 1).

**Table 1:** Characteristics of patients hospitalized with a first episode of thrombotic microangiopathy at the time of diagnosis, according to the etiology of pediatric thrombotic microangiopathy.

Characteristics	Available data	Whole cohort *N* = 303	TTP *n* = 24	STEC-HUS *n* = 14	Secondary TMA *n* = 172	aHUS *n* = 93
Age (y), median (IQR)	303	6.3 (2.4-10.6)	9.1 (3.2-12.5)	2.0 (1.5-3.9)	8.0 (3.8-12.1)	5.4 (1.4-7.2)
Female sex, *n* (%)	303	154 (51)	9 (38)	8 (57)	86 (50)	51 (55)
Organ dysfunction, *n* (%)						
Kidney involvement	303	186 (61)	7 (29)	10 (71)	76 (44)	93 (100)
AKI requiring dialysis	303	76 (25)	2 (8)	2 (14)	37 (22)	35 (38)
Gastrointestinal tract	303	156 (52)	5 (21)	14 (100)	89 (52)	48 (52)
Respiratory system	303	98 (32)	2 (8)	2 (14)	75 (44)	19 (20)
RF requiring invasive mechanical ventilation	303	46 (15)	2 (8)	0 (0)	34 (20)	10 (11)
Central nervous system	303	75 (25)	7 (29)	2 (14)	55 (32)	11 (12)
Seizure	303	48 (16)	3 (13)	2 (14)	35 (20)	8 (9)
Cardiovascular system	303	99 (33)	5 (21)	1 (7)	79 (46)	14 (15)
Heart failure and/or shock	303	39 (13)	1 (4)	1 (7)	31 (18)	6 (7)
HB (g/L), mean ± SD	303	74 ± 20	78 ± 19	73 ± 10	78 ± 21	67 ± 18
PLT (×10^9^/L), median (IQR)	303	34 (17-59)	17 (6-39)	30 (19-43)	35 (17-61)	38 (22-62)
LDH (IU/L), median (IQR)	303	896 (458-2174)	808 (461-1539)	1858 (1122-2298)	528 (368-1051)	2085 (1185-2931)
SCR (mg/dL), median (IQR)	303	0.98 (0.40-2.12)	0.58 (0.29-1.00)	1.48 (0.46-4.54)	0.58 (0.25-1.39)	2.01 (1.08-3.06)
UPCR (g/g), median (IQR)	172	5.65 (2.00-12.18)	0.70 (0.30-9.80)	7.55 (3.03-10.73)	2.70 (1.15-6.90)	10.55 (3.25-20.88)
ADAMTS-13 activity (%), median (IQR)	91	71 (3-93)	0 (0-0.78)	74 (64-78)	73 (53-92)	92 (69-100)
C3 (g/L), mean ± SD	223	0.63 ± 0.28	0.69 ± 0.27	0.71 ± 0.16	0.62 ± 0 .35	0.62 ± 0.18
C5b9 (ng/mL), median (IQR)	90	335 (211-516)	N/A	N/A	329 (206-518)	361 (255-493)

ADAMTS-13, a disintegrin-like and metalloprotease with thrombospondin type 1 motif 13; aHUS, atypical hemolytic uremic syndrome; AKI, acute kidney injury; C3, complement component 3; C5b9, membrane attack complex; HB, hemoglobin; LDH, lactate dehydrogenase; N/A: not available; PLT, platelet; RF, respiratory failure; SCR, serum creatinine; STEC-HUS, Shiga toxin-producing *Escherichia coli*-associated hemolytic uremic syndrome; TMA, thrombotic microangiopathy; TTP, thrombotic thrombocytopenic purpura; UPCR, urine protein-to-creatinine ratio.

### 3.2 Clinical characteristics

Table 1 and Figure 2 describe the clinical and laboratory features of the different etiologic TMA categories. At disease onset, platelet counts in patients with TTP were significantly lower than in those with secondary TMA or aHUS (*P* < .01 and *P* = .02, respectively); serum creatinine concentration in patients with TTP was also significantly lower than in those with aHUS or STEC-HUS (*P* < .001 and *P* = .03, respectively). Compared with TTP and secondary TMA, aHUS was associated with more severe kidney injury and hemolysis at onset, as reflected by significantly higher serum creatinine, lactate dehydrogenase, and urine protein-to-creatinine ratio (all *P* values < .001). Supplementary Table S3 summarizes the missing data on laboratory characteristics.

The clinical manifestations of TMA involved multiple organ systems (Table 1). The proportion of kidney involvement at onset was high in aHUS and STEC-HUS, at 100% and 71%, respectively, with 38% of aHUS patients requiring dialysis during their first hospitalization. All patients with STEC-HUS presented with gastrointestinal symptoms at onset, and half of the aHUS patients also had gastrointestinal symptoms. Secondary TMA presented with multisystem involvement, including gastrointestinal (52%), respiratory (44%), renal (44%), and cardiovascular systems (46%). The proportion of neurologic involvement in TTP (29%) was higher than in STEC-HUS (14%) and aHUS (12%).

The clinical features of secondary TMA caused by common etiologies, including HSCT, systemic lupus erythematosus, cobalamin C deficiency, *Streptococcus pneumoniae*-associated hemolytic uremic syndrome (Sp-HUS), and malignant hypertension, are summarized in Supplementary Table S4. Kidney involvement was highly prevalent in patients with Sp-HUS and malignant hypertension (91% and 86%, respectively), as were those requiring dialysis in Sp-HUS and malignant hypertension (64% and 71%, respectively). Respiratory involvement was also highly prevalent in Sp-HUS, affecting 91% of patients, of whom 64% required mechanical ventilation. All patients with malignant hypertension had cardiovascular involvement, and 28.6% developed heart failure or shock.

### 3.3 Treatments

Table 2 describes the treatments and outcomes based on etiologic classification. Among the 303 patients with TMA, 250 (83%) and 126 (42%) received plasma infusion and plasma exchange therapy, respectively. A total of 72/93 (77%) and 9/24 (38%) patients with aHUS and TTP, respectively, received plasma exchange therapy. Plasma exchange was primarily administered to patients with severe iTTP (9/24 cases). Among the remaining 15 patients, 6 with cTTP received plasma infusion or recombinant ADAMTS-13 therapy, whereas 9 with iTTP (including 2 cases secondary to systemic lupus erythematosus) achieved remission with immunosuppressive therapy (such as corticosteroids and rituximab) combined with plasma infusion, without requiring plasma exchange. Plasma exchange was also used in 4 (29%) patients with STEC-HUS and in 41 (24%) with secondary TMA.

**Table 2:** Treatment and outcomes of pediatric thrombotic microangiopathy.

Treatments and outcomes	Whole cohort *N* = 303	2015.12-2019.12 (*n* = 111)				2020.1-2025.1 (*n* = 192)			
		TTP *n* = 11	STEC-HUS *n* = 11	Secondary TMA *n* = 46	aHUS *n* = 43	TTP *n* = 13	STEC-HUS *n* = 3	Secondary TMA *n* = 126	aHUS *n* = 50
Treatments, *n* (%)									
Plasma transfusion	250 (83)	11 (100)	11 (100)	34 (74)	40 (93)	9 (69)	2 (67)	102 (81)	41 (82)
Plasmapheresis	126 (42)	6 (55)	4 (36)	10 (22)	40 (93)	3 (23)	0 (0)	31 (25)	32 (64)
Eculizumab	39 (13)	0 (0)	0 (0)	0 (0)	0 (0)	0 (0)	0 (0)	15 (12)	24 (48)
Defibrotide	75 (25)	0 (0)	0 (0)	18 (39)	0 (0)	0 (0)	0 (0)	57 (45)	0 (0)
Follow-up time (mo), median (IQR)	35 (12-74)	82 (74-107)	86 (85-89)	72 (4-91)	86 (77-101)	20 (13-46)	22 (8-40)	26 (5-41)	24 (17-36)
Status at last follow-up, *n* (%)									
Alive, no KF	242 (80)	10 (91)	11 (100)	30 (65)	38 (88)	13 (100)	3 (100)	89 (71)	48 (96)
Alive, KF	3 (1)	0 (0)	0 (0)	0 (0)	0 (0)	0 (0)	0 (0)	2 (2)	1 (2)
Dead	58 (19)	1 (9)	0 (0)	16 (35)	5 (12)	0 (0)	0 (0)	35 (28)	1 (2)

aHUS, atypical hemolytic uremic syndrome; KF, kidney failure; STEC-HUS, Shiga toxin-producing *Escherichia coli*-associated hemolytic uremic syndrome; TMA, thrombotic microangiopathy; TTP, thrombotic thrombocytopenic purpura.

Among the 93 patients with aHUS, 24 (26%) were treated with eculizumab. Before eculizumab was launched in China in November 2022, we primarily used traditional plasma infusion or plasma exchange therapy. Eculizumab was also used in 15/172 (9%) patients with secondary TMA. Among the 15 patients with secondary TMA, 6 received eculizumab for HSCT-associated TMA (40%) and 5 for systemic lupus erythematosus-associated TMA (33%). The remaining patients had TMA associated with malignant hypertension, cobalamin C deficiency, or *S pneumoniae* infection. Among patients with secondary TMA, 75 (44%) received defibrotide therapy, all post-HSCT.

Five patients with systemic lupus erythematosus and biopsy-proven TMA, unresponsive to corticosteroids and cyclophosphamide, were treated with eculizumab. Among the 4 responders, eculizumab was initiated approximately 1 week after disease onset, resulting in rapid hematologic normalization (platelet count >150 × 10^9^/L and normalization of lactate dehydrogenase) within 1 to 4 weeks. One patient was able to discontinue dialysis, and another showed resolution of central nervous system symptoms. The fifth patient initially received plasma exchange and rituximab, with eculizumab initiated in the fourth week. Although platelet recovery occurred after 3 doses of eculizumab, the patient progressed to permanent dialysis dependence despite rescue therapy.

Among the 6 pediatric HSCT patients treated with eculizumab, the underlying diseases included acute lymphoblastic leukemia, non-Hodgkin lymphoma, severe aplastic anemia, juvenile myelomonocytic leukemia, mucopolysaccharidosis type II, and severe combined immunodeficiency. Five of these patients were diagnosed with TMA within 3 months after transplantation and received 3 to 6 doses of eculizumab. All were assessed as nonresponders at the end of treatment or at the time of death, and 3 ultimately died. The remaining patient with mucopolysaccharidosis type II was diagnosed with TMA 280 days after transplantation and achieved control of hemolysis after 2 doses of eculizumab.

### 3.4 Analysis of risk factors for outcomes and prognosis

During a median follow-up of 35 months (range, 0.1-114.6), 61 (20%) patients died or progressed to kidney failure (Table 2). Patients with secondary TMA had the worst outcomes, with 31% (53/172) experiencing death or kidney failure, whereas no deaths or kidney failure occurred in STEC-HUS patients. Among those with secondary TMA, the proportion of patients who died or progressed to kidney failure was 36% (34/95) for HSCT and 31% (5/16) for cobalamin C deficiency (Supplementary Table S4). Patients with aHUS and TTP showed moderate composite outcomes (death or kidney failure at 6/93 [7%] and 1/24 [4%], respectively). All 7 aHUS patients who died or progressed to kidney failure were from before the eculizumab era. However, the proportion of kidney failure or death was higher in secondary TMA, whether between December 2015 and December 2019 or between January 2020 and January 2025 (35% and 30%, respectively), than in other TMA subgroups.

We further analyzed the relationship between the involvement of different systems and the occurrence of composite outcomes (Supplementary Figure S1A). At onset, patients presenting with respiratory, central nervous, or cardiovascular system manifestations had a significantly higher proportion of death or progression to kidney failure compared with those without relevant system involvement (36% vs 13%, 37% vs 15%, 36% vs 12%; all *P* values < .001). Further analysis showed that the main respiratory manifestation was invasive mechanical ventilation, the neurologic manifestation was seizures, and cardiovascular manifestations were heart failure and/or shock, all of which were risk factors for death or progression to kidney failure (Supplementary Figure S1B).

**Figure 3:**
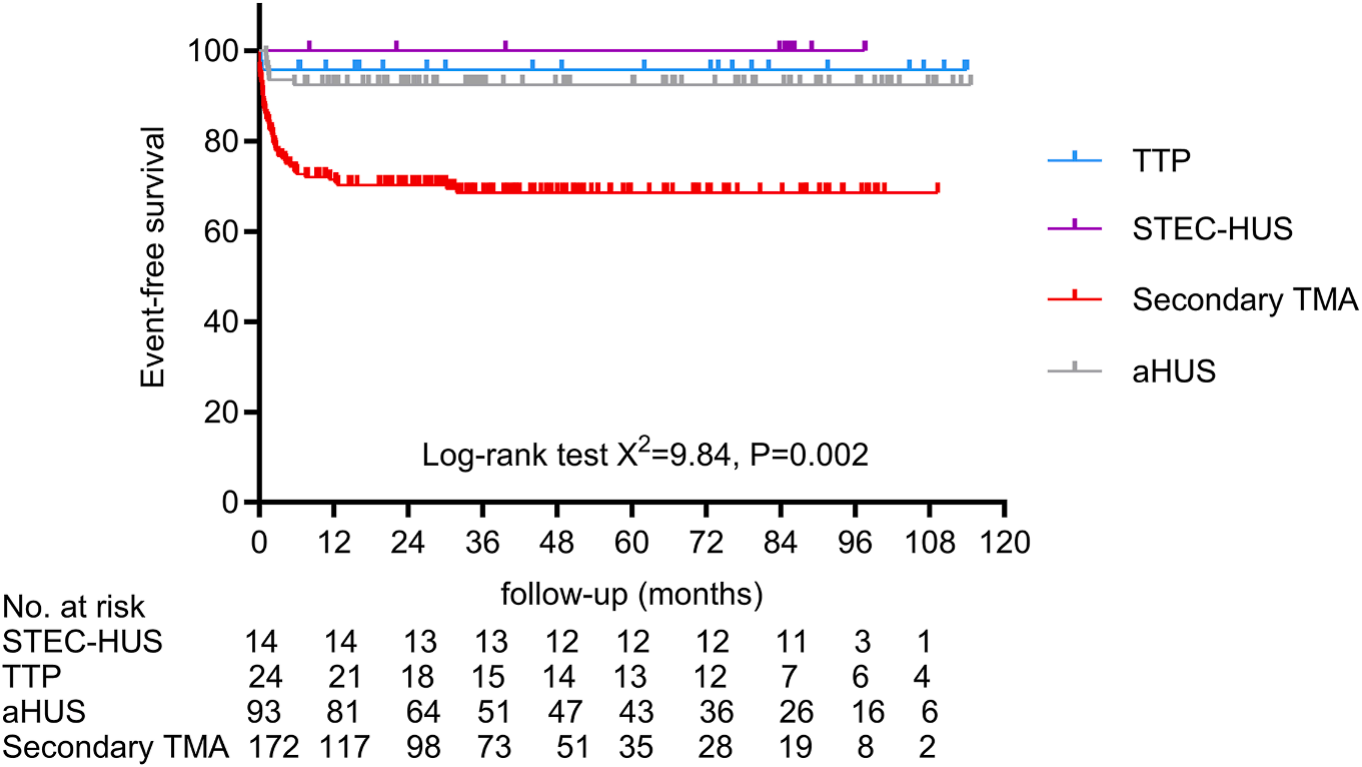
Kaplan–Meier survival-free curves for kidney failure and survival, according to thrombotic microangiopathy (TMA) etiology. aHUS, atypical hemolytic uremic syndrome; STEC-HUS, Shiga toxin-producing *Escherichia coli*-associated hemolytic uremic syndrome; TTP, thrombotic thrombocytopenic purpura.

Table 3 shows the Cox regression models used to develop composite outcomes. Compared with aHUS patients, those with secondary TMA had a poorer prognosis, in both the univariate analysis (HR, 4.67; 95% CI, 2.12-10.27; *P* < .001) and the multivariate analysis (HR, 3.57; 95% CI, 1.53-8.30; *P* < .01). Figure 3 shows the Kaplan–Meier analysis of event-free survival (no death or kidney failure) across the different etiologic categories of TMA. Patients with secondary TMA had significantly lower 9-year event-free survival than those without secondary TMA (chi-square test = 9.84; *P* = .002).

**Table 3:** Cox regression analyses of kidney failure or death.

Variables	Univariate Cox regression			Multivariate Cox regression model		
	HR	95% CI	*P*	HR	95% CI	*P*
Age (y)	1.03	0.98-1.09	.26			
Female sex	1.25	0.75-2.07	.39			
AKI requiring dialysis	0.75	0.43-1.29	.30			
RF requiring invasive mechanical ventilation	4.98	2.97-8.34	<.001	3.88	2.19-6.88	.001
Seizure	2.14	1.21-3.79	.01	0.86	0.45-1.64	.65
Heart failure and/or shock	3.60	2.08-6.25	<.001	2.21	1.24-3.93	.01
HB (g/L)	1.01	0.99-1.02	.38			
PLT (10^3^/μL)	0.99	0.99-1.00	.12			
LDH (U/L)	1.00	1.00-1.00	.45			
SCR (mg/dL)	0.82	0.67-1.01	.06	0.95	0.75-1.21	.70
UPCR (mg/mg)	1.01	0.98-1.04	.42			
C3 (g/L)	1.29	0.35-4.78	.70			
Plasma transfusion	1.10	0.56-2.17	.78			
Plasmapheresis	0.67	0.39-1.15	.15			
Eculizumab	0.60	0.24-1.49	.27			
Defibrotide	2.73	1.65-4.52	<.001	1.23	0.65-2.32	.53
Etiology of TMA						
aHUS	1.00					
TTP	0.56	0.07-4.53	.58	0.66	0.08-5.51	.70
STEC-HUS	0.00	0.00-6.56E + 210	.97	0.00	0.00-1.99E + 235	.98
Secondary TMA	4.67	2.12-10.27	<.001	3.57	1.53-8.30	<.01

aHUS, atypical hemolytic uremic syndrome; AKI, acute kidney injury; C3, complement component 3; HB, hemoglobin; HR, hazard ratio; LDH, lactate dehydrogenase; PLT, platelet; RF, respiratory failure; SCR, serum creatinine; STEC-HUS, Shiga toxin-producing *Escherichia coli*-associated hemolytic uremic syndrome; TMA, thrombotic microangiopathy; TTP, thrombotic thrombocytopenic purpura; UPCR, urine protein-to-creatinine ratio.

Table 3 presents the parameters of the univariate Cox proportional hazards regression model evaluating the ability of clinical presentation, laboratory tests, and treatment variables to predict renal survival, as well as the multivariate Cox model adjusted for clinical and laboratory features. Clinical presentations of heart failure and/or shock at disease onset (HR, 2.21; 95% CI, 1.24-3.93; *P* = .01) and the need for mechanical ventilation due to respiratory failure (HR, 3.88; 95% CI, 2.19-6.88; *P* < .001) were also potential risk factors for adverse renal outcomes in children.

## 4 Discussion

In this study, we screened more than 600,936 patients at the National Children’s Medical Center in China over a period of 9 years and identified 303 consecutive patients hospitalized with a first episode of TMA. We observed that secondary TMA accounted for more than half of the cases of pediatric TMA (approximately 57%), with significantly higher involvement of the respiratory and cardiovascular systems compared with other TMA subgroups, and was associated with a high risk of death or progression to kidney failure. Secondary TMA, the presence of heart failure and/or shock at onset, and the need for mechanical ventilation due to respiratory failure were independent risk factors for death or progression to kidney failure in children with TMA.

TMA is a rare condition at our center, representing a very small proportion (0.05%) of the total patient population. Notably, as a tertiary pediatric referral center, the etiologic distribution of TMA in our cohort was markedly skewed, with secondary forms (predominantly driven by HSCT and systemic lupus erythematosus) constituting the vast majority of cases. This patient profile suggests that our findings primarily reflect a severe and complex subgroup of TMA.

The high prevalence of secondary TMA in our cohort is consistent with recent studies in Europe (France, Germany, and Belgium) that enrolled patients across all age groups, in which secondary TMA accounted for 49% to 88% of all TMAs [24,32,33]. However, pediatric patients constituted only 3% to 25% of the cohorts in these studies (Supplementary Table S1). In contrast, in an Asian pediatric TMA cohort [30], Ashida et al. [30] reported that secondary TMA accounted for only 10% of 258 Japanese children with TMA. That study [30] enrolled patients through 2 nationwide cross-sectional surveys rather than consecutive recruitment, and differences in inclusion criteria may partly explain the discrepancies in etiologic classification. Subsequently, in 2020, Katsuno et al. [34] enrolled 152 TMA patients through the Japan Renal Biopsy Registry, of whom 49% had secondary TMA.

The proportion of STEC-HUS in the TMA cohort across all age groups ranges from 1% to 21% [24,32,33,35], whereas a notably higher proportion (64%) was reported in a Japanese pediatric TMA cohort, which did not recruit patients consecutively [30]. In our study, the proportion of STEC-HUS was only 5%. Because our center is a national medical center that receives complex, severe, or diagnostically challenging TMA cases, some patients may recover quickly after receiving supportive treatment at primary hospitals and therefore may not require transfer to our center. This may partially explain the low proportion of STEC-HUS in this study.

The etiology of secondary TMA varies widely across studies, reflecting differences in inclusion criteria and professional expertise among different centers [23,24,30,[32], [33], [34]]. In this study, HSCT and systemic diseases (mainly systemic lupus erythematosus) were the leading causes of secondary TMA, accounting for 77% of all cases.

Consistent with other studies [33], secondary TMA was associated with the highest risk of death or kidney failure. This study showed that patients with secondary TMA presented with multiple organ involvement at onset, and the proportion of patients with respiratory, central nervous, or cardiovascular system involvement was significantly higher than in those without related system involvement in terms of poor prognosis. This paragraph outlines several factors contributing to the poor prognosis in patients with secondary TMA. In addition, due to the complex etiology, delayed diagnosis, and lack of specific treatment, secondary TMA can only be treated with targeted supportive therapy for various systemic symptoms. Our study suggests that secondary TMA is likely the predominant subtype encountered in comprehensive tertiary pediatric centers and is associated with unfavorable outcomes. Therefore, future research needs to strengthen early diagnosis and explore the pathogenesis of secondary TMA.

Our research has several strengths. We included consecutive patients without selection bias and reported clinical features and long-term outcomes for a subset of patients with secondary TMA. We also need to acknowledge several limitations, including the retrospective, single-center study design, the lack of systematic complement testing to assess complement status, and the absence of screening for complement gene variations.

In conclusion, by examining a large number of consecutive TMA patients, this study identified secondary TMA as the main cause of pediatric TMA. Secondary TMA, clinical manifestations of heart failure and/or shock at onset, and the need for mechanical ventilation due to respiratory failure are independent risk factors for death or kidney failure in children with TMA. Future research is needed to better understand the mechanisms underlying multiorgan damage in patients with secondary TMA and to improve prognosis.

## References

[1] Fakhouri F., Frémeaux-Bacchi V. (2021). Thrombotic microangiopathy in aHUS and beyond: clinical clues from complement genetics. Nat Rev Nephrol.

[2] Goodship T.H., Cook H.T., Fakhouri F., Fervenza F.C., Frémeaux-Bacchi V., Kavanagh D. (2017). Atypical hemolytic uremic syndrome and C3 glomerulopathy: conclusions from a “Kidney Disease: Improving Global Outcomes” (KDIGO) Controversies Conference. Kidney Int.

[3] Zheng X.L., Vesely S.K., Cataland S.R., Coppo P., Geldziler B., Iorio A. (2020). ISTH guidelines for the diagnosis of thrombotic thrombocytopenic purpura. J Thromb Haemost.

[4] Claes K.J., Massart A., Collard L., Weekers L., Goffin E., Pochet J.M. (2018). Belgian consensus statement on the diagnosis and management of patients with atypical hemolytic uremic syndrome. Acta Clin Belg.

[5] Zheng X.L. (2015). ADAMTS13 and von Willebrand factor in thrombotic thrombocytopenic purpura. Annu Rev Med.

[6] Graça N.A.G., Joly B.S., Voorberg J., Vanhoorelbeke K., Béranger N., Veyradier A. (2022). TTP: from empiricism for an enigmatic disease to targeted molecular therapies. Br J Haematol.

[7] Scully M., Cataland S.R., Peyvandi F., Coppo P., Knöbl P., Kremer Hovinga J.A. (2019). Caplacizumab treatment for acquired thrombotic thrombocytopenic purpura. N Engl J Med.

[8] Scully M., Antun A., Cataland S.R., Coppo P., Dossier C., Biebuyck N. (2024). Recombinant ADAMTS13 in congenital thrombotic thrombocytopenic purpura. N Engl J Med.

[9] Fakhouri F., Zuber J., Fremeaux-Bacchi V., Loirat C. (2017). Haemolytic uraemic syndrome. Lancet.

[10] Schwartz J., Padmanabhan A., Aqui N., Balogun R.A., Connelly-Smith L., Delaney M. (2016). Guidelines on the use of therapeutic apheresis in clinical practice-evidence-based approach from the writing committee of the American Society for Apheresis: the seventh special issue. J Clin Apher.

[11] Menne J., Nitschke M., Stingele R., Abu-Tair M., Beneke J., Bramstedt J. (2012). Validation of treatment strategies for enterohaemorrhagic Escherichia coli O104:H4 induced haemolytic uraemic syndrome: case-control study. BMJ.

[12] Freedman S.B., Xie J., Neufeld M.S., Hamilton W.L., Hartling L., Tarr P.I. (2016). Shiga toxin-producing Escherichia coli infection, antibiotics, and risk of developing hemolytic uremic syndrome: a meta-analysis. Clin Infect Dis.

[13] Palomo M., Blasco M., Molina P., Lozano M., Praga M., Torramade-Moix S. (2019). Complement activation and thrombotic microangiopathies. Clin J Am Soc Nephrol.

[14] Zuber J., Frimat M., Caillard S., Kamar N., Gatault P., Petitprez F. (2019). Use of highly individualized complement blockade has revolutionized clinical outcomes after kidney transplantation and renal epidemiology of atypical hemolytic uremic syndrome. J Am Soc Nephrol.

[15] Rondeau E., Scully M., Ariceta G., Barbour T., Cataland S., Heyne N. (2020). The long-acting C5 inhibitor, Ravulizumab, is effective and safe in adult patients with atypical hemolytic uremic syndrome naïve to complement inhibitor treatment. Kidney Int.

[16] George J.N., Nester C.M. (2014). Syndromes of thrombotic microangiopathy. N Engl J Med.

[17] Khan S., George J.N., Nusrat S. (2023). Drug-induced thrombotic microangiopathy and thrombotic thrombocytopenic purpura: a systematic review, 2018-2023. Am J Hematol.

[18] Merzkani M., Chann Wai Lynn N., Flores K., Rajashekar G., Progar K., Santos R.D. (2025). Thrombotic microangiopathy after kidney transplantation: insights into genetic etiology and clinical outcomes. Kidney Int Rep.

[19] Luckie T., Wahba A., Al-Khatib H., Fernandez Sanchez J., Salem B., Srivaths P. (2025). Characteristics and outcomes of pediatric hematopoietic stem cell transplantation-associated thrombotic microangiopathy: a retrospective single-center analysis. Blood.

[20] Java A., Kim A.H.J. (2023). The role of complement in autoimmune disease-associated thrombotic microangiopathy and the potential for therapeutics. J Rheumatol.

[21] Scheiring J., Andreoli S.P., Zimmerhackl L.B. (2008). Treatment and outcome of Shiga-toxin-associated hemolytic uremic syndrome (HUS). Pediatr Nephrol.

[22] Noris M., Remuzzi G. (2009). Atypical hemolytic-uremic syndrome. N Engl J Med.

[23] Henry N., Mellaza C., Fage N., Beloncle F., Genevieve F., Legendre G. (2021). Retrospective and systematic analysis of causes and outcomes of thrombotic microangiopathies in routine clinical practice: an 11-year study. Front Med (Lausanne).

[24] Bayer G., von Tokarski F., Thoreau B., Bauvois A., Barbet C., Cloarec S. (2019). Etiology and outcomes of thrombotic microangiopathies. Clin J Am Soc Nephrol.

[25] Jodele S., Davies S.M., Lane A., Khoury J., Dandoy C., Goebel J. (2014). Diagnostic and risk criteria for HSCT-associated thrombotic microangiopathy: a study in children and young adults. Blood.

[26] Gai Y., Li M., Zhu Z., Zhou Y., Huang C., Bai W. (2025). Systematic review of the outcomes and prognostic factors of patients with systemic lupus erythematosus-associated thrombotic microangiopathy. Kidney Int Rep.

[27] Minoia F., Tibaldi J., Muratore V., Gallizzi R., Bracaglia C., Arduini A. (2021). Thrombotic microangiopathy associated with macrophage activation syndrome: a multinational study of 23 patients. J Pediatr.

[28] Fremeaux-Bacchi V., Fakhouri F., Garnier A., Bienaimé F., Dragon-Durey M.A., Ngo S. (2013). Genetics and outcome of atypical hemolytic uremic syndrome: a nationwide French series comparing children and adults. Clin J Am Soc Nephrol.

[29] Massart A., Weekers L., Claes K.J., Van Meerhaeghe T., Snauwaert E., Mekahli D. (2025). Clinical and genetic characteristics of patients diagnosed with atypical hemolytic uremic syndrome (aHUS): epidemiological data from the Belgian cohort of the Global aHUS Registry. J Nephrol.

[30] Ashida A., Matsumura H., Sawai T., Fujimaru R., Fujii Y., Shirasu A. (2018). Clinical features in a series of 258 Japanese pediatric patients with thrombotic microangiopathy. Clin Exp Nephrol.

[31] Singbartl K., Kellum J.A. (2012). AKI in the ICU: definition, epidemiology, risk stratification, and outcomes. Kidney Int.

[32] Schönermarck U., Ries W., Schröppel B., Pape L., Dunaj-Kazmierowska M., Burst V. (2020). Relative incidence of thrombotic thrombocytopenic purpura and haemolytic uraemic syndrome in clinically suspected cases of thrombotic microangiopathy. Clin Kidney J.

[33] Werion A., Storms P., Zizi Y., Beguin C., Bernards J., Cambier J.F. (2023). Epidemiology, outcomes, and complement gene variants in secondary thrombotic microangiopathies. Clin J Am Soc Nephrol.

[34] Katsuno T., Ito Y., Kagami S., Kitamura H., Maruyama S., Shimizu A. (2020). A nationwide cross-sectional analysis of thrombotic microangiopathy in the Japan Renal Biopsy Registry (J-RBR). Clin Exp Nephrol.

[35] Dos Santos C., Paiva J., Romero M.L., Agazzoni M., Kempfer A.C., Rotondo S. (2021). Thrombotic microangiopathies: first report of 294 cases from a single institution experience in Argentina. EJHaem.

