# Supplementary material for: Analysis of causes and outcomes of principal secondary thrombotic microangiopathy: a 9-year cohort study from a tertiary pediatric center in China
Source: Res Pract Thromb Haemost. 2026 Jul 14;10(5):106850. doi: 10.1016/j.rpth.2026.106850 (PMC13452974; doi:10.1016/j.rpth.2026.106850)
Supplement: Supplementary file 1 [file mmc1.docx]

**Supplemental Table 1.** Comparison of cohort studies of TMA.

| Author, year | Akira,  2018 | Bayer,  2019 | Schönermarck, 2020 | Dos Santos, 2020 | Takayuki,  2020 | Werion,  2023 | Wu,  Current study |
| --- | --- | --- | --- | --- | --- | --- | --- |
| Patients with TMA, n | 258 | 564 | 232 | 294 | 152 | 336 | 303 |
| Number of children, % | 100 | 3 | 6 | 25^*^ | 9 | 25^*^ | 100 |
| Secondary TMA, n (%) | 27(10) | 496 (88) | 98 (42) | 46(16) | 75(49) | 188 (56) | 172 (57) |
| STEC-HUS, n (%) | 166(64) | 35(6) | 15(6) | 3(1) | N/A | 70(21) | 14 (5) |
| aHUS, n (%) | 40(16) | 15(3) | 55(24) | 94(32) | N/A | 49(15) | 93 (31) |
| TTP, n (%) | 15(6) | 18(3) | 31(13) | 72(24) | N/A | 29(9) | 24 (8) |
| Study design | Multicentric, retrospective | Multicentric, retrospective | Multicentric, prospective | Multicentric, retrospective | Multicentric, retrospective | Multicentric, retrospective | Monocentric, retrospective |
| Inclusion criteria | A cross-sectional nationwide survey | Consecutive patients with TMA | Consecutive patients with TMA | Consecutive patients with TMA | The Japan Renal Biopsy Registry (J-RBR) | Consecutive patients with TMA | Consecutive patients with TMA |
| Long term follow-up | No | No | No | No | No | Yes | Yes |

TMA, thrombotic microangiopathy; STEC-HUS, Shiga toxin–associated hemolytic uremic syndrome; aHUS, atypical hemolytic uremic syndrome; TTP, thrombotic thrombocytopenic purpura; N/A, not available.

^*^: Data are approximate.

**Supplemental Table 2.** Etiologies of secondary TMA.

| Diagnosis | no. (%) |
| --- | --- |
| Hematopoietic stem cell transplantation | 95 (55) |
| Systemic diseases^*^ | 40 (23) |
| Cobalamin C deficiency | 16 (9) |
| Infections^#^ | 12 (7) |
| Malignant hypertension | 7 (4) |
| Neoplasia^&^ | 2 (1) |

^*^Systemic diseases include systemic lupus erythematosus (38) and macrophage activation syndrome (2). ^#^Infections include Streptococcus pneumoniae infection (11) and Epstein-Barr virus infection (1). ^&^Neoplasia include 2 cases of non-Hodgkin lymphoma. TMA, thrombotic microangiopathy.

**Supplemental Table 3.** Information about available and missing data of baseline.

| Available/missing data | Whole cohort  n=303 | TTP  n=24 | STEC-HUS  n=14 | Secondary TMA  n=172 | aHUS  n=93 |
| --- | --- | --- | --- | --- | --- |
| Age | 303/0 | 24/0 | 14/0 | 172/0 | 93/0 |
| Hb | 303/0 | 24/0 | 14/0 | 172/0 | 93/0 |
| PLT | 303/0 | 24/0 | 14/0 | 172/0 | 93/0 |
| LDH | 303/0 | 24/0 | 14/0 | 172/0 | 93/0 |
| SCR | 303/0 | 24/0 | 14/0 | 172/0 | 93/0 |
| UPCR | 172/131 | 5/19 | 14/0 | 77/95 | 76/17 |
| ADAMTS13 activity | 91/212 | 24/0 | 5/9 | 25/147 | 37/56 |
| C3 | 223/80 | 18/6 | 14/0 | 101/71 | 90/3 |
| C5b9 | 90/213 | 3/21 | 0/14 | 77/95 | 10/83 |

TTP, thrombotic thrombocytopenic purpura; STEC-HUS, Shiga toxin–associated hemolytic uremic syndrome; aHUS, atypical hemolytic uremic syndrome; HB, hemoglobin; PLT, platelets; LDH, lactate dehydrogenase; SCR, serum creatinine; UPCR, urine protein-to-creatinine ratio; ADAMTS13, a disintegrin-like and metalloprotease with thrombospondin type 1 motif 13; C3, complement component 3; C5b9, membrane attack complex.

**Supplemental Table 4.** Baseline characteristics, treatment, and outcomes of patients with the main etiologies of secondary TMA

| Characteristics | Secondary TMA  n=172 | HSCT  n=95 | SLE  n=38 | Cobalamin C deficiency  n=16 | SP-HUS  n=11 | Malignant hypertension  n=7 |
| --- | --- | --- | --- | --- | --- | --- |
| Age, median (IQR), years | 8.0(3.8–12.1) | 7.4 (4.1–11.0) | 12.6(10.9–14.4) | 2.7(0.3–5.1) | 2.9 (1.6–3.5) | 4.8(1.2–13.4) |
| Female gender, n (%) | 86(50.0) | 61(64.2) | 3(7.9) | 10(62.5) | 5(45.5) | 5(71.4) |
| Organ dysfunction, n (%) |  |  |  |  |  |  |
| Kidney involvement | 76(44.2) | 13(13.7) | 30(78.9) | 14(87.5) | 10(90.9) | 6(85.7) |
| AKI requiring dialysis | 37(21.5) | 2(2.1) | 11(28.9) | 9(56.3) | 7(63.6) | 5(71.4) |
| Gastrointestinal tract | 89(51.7) | 61(64.2) | 9(23.7) | 9(56.3) | 6(54.5) | 4(57.1) |
| Respiratory system | 75(43.6) | 28(29.5) | 25(65.8) | 8(50.0) | 10(90.9) | 3(42.9) |
| RF requiring invasive mechanical ventilation | 34(19.8) | 17(17.9) | 2(5.3) | 5(31.3) | 7(63.6) | 2(28.6) |
| Central nervous system | 55(32.0) | 30(31.6) | 11(28.9) | 6(37.5) | 4(36.4) | 2(28.6) |
| Seizure | 35(20.3) | 20(21.1) | 5(13.2) | 5(31.3) | 2(18.2) | 1(14.3) |
| Cardiovascular system | 79(45.9) | 33(34.7) | 22(57.9) | 10(62.5) | 4(36.4) | 7(100.0) |
| Heart failure and/or Shock | 31(18.0) | 16(16.8) | 6(15.8) | 3(18.8) | 1(9.1) | 2(28.6) |
| HB, mean ± SD, g/L | 78±21 | 83±18 | 75±21 | 67±17 | 64±27 | 68±15 |
| PLT, median (IQR), x10^9^/L | 35(17–61) | 29(16–48) | 45(24–78) | 85(28–126) | 14 (7–50) | 78(56–102) |
| LDH, median (IQR), IU/L | 528(368–1051) | 482(367–698) | 381(339–679) | 1568(874–2373) | 2625 (1174–5155) | 781(587–1531) |
| SCR, median (IQR), mg/dL | 0.58(0.25-1.39) | 0.29(0.18–0.53) | 1.43(1.16–2.67) | 0.93(0.54–1.89) | 2.11 (1.01–2.95) | 1.83(0.86–5.42) |
| UPCR, median (IQR), g/g | 2.70(1.15-6.90) | 1.20(0.58–2.10) | 5.40(2.60–7.05) | 8.40(2.30–15.10) | N/A | 3.95(2.40–14.75) |
| C3, mean ± SD, g/L | 0.62±0.35 | 0.88±0.31 | 0.34±0.25 | 0.60±0.14 | 0.69±0.34 | 0.81±0.21 |
| C5b9, median (IQR), ng/ml | 329(206-518) | 282(195–373) | 988(516–1167) | N/A | N/A | N/A |
| Treatments, n (%) |  |  |  |  |  |  |
| Plasma transfusion | 136(79.1) | 78(82.1) | 30(78.9) | 12(75.0) | 8(72.7) | 5(71.4) |
| Plasmapheresis | 41(23.8) | 7(7.4) | 20(52.6) | 6(37.5) | 5(45.5) | 1(14.3) |
| Eculizumab | 15(8.7) | 6(6.3) | 5(13.2) | 1(6.3) | 1(9.1) | 2(28.6) |
| Defibrotide | 75(43.6) | 75(78.9) | 0 | 0 | 0 | 0 |
| Follow-up time, median (IQR), months | 30(5–53) | 33 (4–59) | 26(7–34) | 27(5–48) | 77 (24–99) | 9(1–41) |
| Status at last follow-up, n (%) |  |  |  |  |  |  |
| Alive, No KF | 119(69) | 61(64) | 30(79) | 11(69) | 10(91) | 4(57) |
| Alive, KF | 2(1) | 0 | 1(3) | 1(6) | 0 | 0 |
| Dead | 51(30) | 34(36) | 7(18) | 4(25) | 1(9) | 3(43) |

TMA, thrombotic microangiopathy; HSCT, hematopoietic stem cell transplantation; SLE, systemic Lupus Erythematosus; SP-HUS, Streptococcus pneumoniae-associated hemolytic uremic syndrome; AKI, acute kidney injury; RF, respiratory failure; HB, hemoglobin; PLT, platelets; LDH, lactate dehydrogenase; SCR, serum creatinine; UPCR, urine protein-to-creatinine ratio; ADAMTS13, a disintegrin-like and metalloprotease with thrombospondin type 1 motif 13; C3, complement component 3; C5b9, membrane attack complex; KF, kidney failure; N/A, not available.

**Supplemental Figure 1.** Analysis of death or kidney failure according to clinical manifestations.

KF, Kidney failure. *P < 0.05; **P < 0.01; ***P < 0.001.
